# Upper extremity muscle thickness, handgrip strength, manual dexterity and cognitive functions in older adults living in community and nursing homes : a cross-sectional study

**DOI:** 10.1038/s41598-026-63937-7

**Published:** 2026-07-24

**Authors:** Süleyman Korkusuz, Büşra Seçkinoğulları Korkusuz

**Affiliations:** 1 Faculty of Health Sciences, Department of Physical Therapy and Rehabilitation, Atılım University, Ankara, Turkey; 2 Kızılcahamam Vocational School of Health Services, Department of Therapy and Rehabilitation, Ankara University, Ankara, Turkey

**Keywords:** Community-dwelling older adults, Nursing home, Upper extremity, Muscle thickness, Hand grip strength, Pinch strength, Manual dexterity, Cognitive function, Health care, Health occupations, Medical research

## Abstract

This study aims to compare upper extremity muscle thickness, hand grip strength, manual dexterity, and cognitive functions in elderly individuals living in the community and nursing homes. A total of 85 older adults with similar demographic characteristics, aged 65–80 years, 39 living in nursing homes and 46 community-dwelling older adults, were included in a cross-sectional study. Hand grip strength (HGS) was assessed via the Jamar hand dynamometer, and pinch strength was assessed via the Jamar digital pinch gauge. The Purdue Pegboard Test (PPT) was used to assess manual dexterity, and the Stroop T-Bag Test was used to assess cognitive function. In addition, upper extremity muscle thickness was assessed via ultrasonography. The forearm, biceps brachii, triceps brachii and deltoid muscle thicknesses were lower in elderly people living in nursing homes than in adults living in the community (p < 0.05). HGS and pinch strength were greater in adults living in the community than in adults living in nursing homes (p < 0.05). The total PPT score was higher in adults living in the community than in adults living in nursing homes (p < 0.05). The MOCA score and Stroop test composite score of elderly individuals living in nursing homes were worse than those of adults living in the community (p < 0.05). In this study, older adults living in nursing homes were observed to have lower upper extremity muscle thickness, handgrip strength, pinch strength, manual dexterity, and cognitive performance compared with community-dwelling older adults. This may be related to differences in living conditions, social resources, and environmental factors between the two groups. These findings suggest that maintaining upper extremity muscle thickness and strength, manual dexterity, and cognitive functions may be important for older adults living in nursing homes.

## Introduction

Aging is a biological process associated with functional decline in which changes in morphological and physiological parameters that become progressive and negative over time, combined with accompanying pathological conditions, cause a decrease in the physical and mental capacity of the individual^1,2^. Decreasing birth rates and increasing life expectancies are leading to global aging^3^. According to World Health Organization (WHO) data, the number of people aged 60 and over, which constituted 12% of the global population in 2015, is expected to increase to 22%, or approximately 2.1 billion people, by 2050^4^. According to Turkish Statistical Institute (TurkStat) data, as of 2021, there were 12 million people aged 60 and over in Türkiye, representing 14% of the total population and increasing annually. Looking at the housing situation of older adults in Türkiye, approximately 1.5 million elderly people live alone, while only 64,000 benefit from institutional services such as nursing homes or care centers^5^.

Environmental factors affecting the functional health of older adults are becoming increasingly important. In this context, the environments in which older individuals reside stand out as determinants that directly impact their daily routines and, consequently, their cognitive and physical abilities. While individuals living in their own homes benefit from the sense of belonging and security brought about by a familiar environment, they also face the mental and physical demands of housework and other daily responsibilities^6,7^. On the other hand, institutional care settings such as nursing homes, which may be preferred for reasons such as balance problems, the need for socialization, or increased care needs, offer older adults a life where their health is better managed, household responsibilities are reduced, and a wide range of social opportunities are offered. However, while this may lead to a decline in physical capacity due to the reduced household responsibilities in nursing homes, the social and recreational activities offered can help maintain physical capacity^6,7^.

In older individuals, reduced physiological capacity and increased sensitivity to external stressors manifest as physical weakness as well as cognitive dysfunction ranging from mild cognitive decline to advanced dementia^8^. The decline in cognitive function may negatively affect daily task performance, limit active movement capacity, and even contribute to muscle weakness, further increasing functional loss. These aging-related deficiencies increase dependency in daily life activities and may lead to various psychological and social problems^9^. Therefore, evaluating cognitive functions in older adults is not only important for mental health but also for understanding their role in maintaining independence and physical capacity.

In addition to cognitive decline, muscle mass and strength also decrease with age. Handgrip strength (HGS) is a common and standardized indicator of overall muscle strength, while ultrasonography (US) provides high accuracy and specificity in determining regional and total muscle mass^10,11^. Accordingly, combining muscle thickness assessment with HGS measurements offers a more comprehensive evaluation of age-related changes in muscle function.

Among the many detrimental effects of aging, deterioration of manual dexterity is a condition that can lead to difficulty performing instrumental activities of daily living, such as writing, cooking, gardening, crafts, and bottle opening^12^. Preserving manual dexterity is crucial for independent daily living in older adults^13^. Literature indicates that lower extremity movements are governed by lower-level central generators, whereas manual dexterity and fine motor control require direct cortical motor control, which is closely associated with higher-level cognitive areas most sensitive to the effects of aging, such as executive function^14,15^. Upper extremity function represents both a sensitive marker for the early detection of cognitive decline and a vital priority for basic ADLs that maintain the independence of older individuals^15,16^. Therefore, to better understand functional capacity in older adults, it is of great importance to evaluate upper extremity muscle thickness, grip and pinch strength, and manual dexterity.

The need to preserve the functional capacity of the elderly population, which increases with increasing human lifespan, forms the basis of this study. The literature indicates that elderly individuals living in nursing homes have higher rates of sarcopenia^17–19^, lower handgrip strength^20,21^, poorer performance on agility and balance tests^6^, and lower levels of physical activity^22^ compared to those living in the community. Similarly, differences have been reported between these two groups in terms of pinch strength^21^ and cognitive functions^23^. However, to our knowledge, no studies have compared upper extremity muscle thickness and manual dexterity between older adults living in nursing homes and older adults living in the community, and there are limited studies examining differences in handgrip strength and cognitive function.

Understanding the diverse effects of environmental factors on functioning and how they affect older adults in different settings, such as those living in institutions or the community, is important for developing strategies that enhance their functioning and overall well-being and enable them to live safely and independently^24^. However, existing studies have not fully elucidated the effects of environmental factors on the functional and cognitive abilities of older adults. Therefore, our study aims to compare the effects of different living environments on upper extremity muscle thickness, handgrip and pinch strength, manual dexterity, and cognitive functions. The findings will guide future intervention programs and health policies aimed at improving the independence and quality of life of elderly individuals.

The hypotheses of this study are:

H1: Older adults living in nursing homes have less upper extremity muscle thickness compared to community-dwelling older adults.

H2: Older adults living in nursing homes have lower hand grip and pinch strength compared to community-dwelling older adults.

H3: Older adults living in nursing homes have weaker manual dexterity compared to community-dwelling older adults.

H4: Older adults living in nursing homes have lower cognitive function compared to community-dwelling older adults.

## Methods

### Study design

A total of 85 older adults were included in the study: 39 older adults living in a nursing home and 46 community-dwelling older adults with similar ages, body mass indices, and exercise habits. The study was conducted at Atılım University and a private nursing home. Participant recruitment and data collection were conducted between June and July 2025. The Human Research Ethics Board of Atılım University approved the study with code 604.01.02–419. The study had a cross-sectional design and was registered at ClinicalTrials.gov on 16 May 2025 after ethics committee approval (NCT06988657). Informed consent was obtained from all participants, and the study was conducted by the Declaration of Helsinki. This study conforms to all STROBE guidelines and reports the required information accordingly.

To avoid bias in the analysis results, the inclusion and exclusion criteria were carefully determined to ensure that the participants had similar characteristics. The study included older adults aged 65–80 years who scored 21 or higher on the Montreal Cognitive Assessment Scale (MoCA) and were able to stand unassisted for at least 90 s. For older adults living in a nursing home, an additional criterion was a minimum of 6 months of nursing home residence. Older adults who were unable to communicate verbally, used walking aids, had severe visual impairment; had sensory disorders such as neuropathy, neurological disorders and/or congestive heart failure; and for whom participation in exercise was not recommended by a physician due to medical contraindications were excluded from the study.

### Sample size

**Fig. 1:**
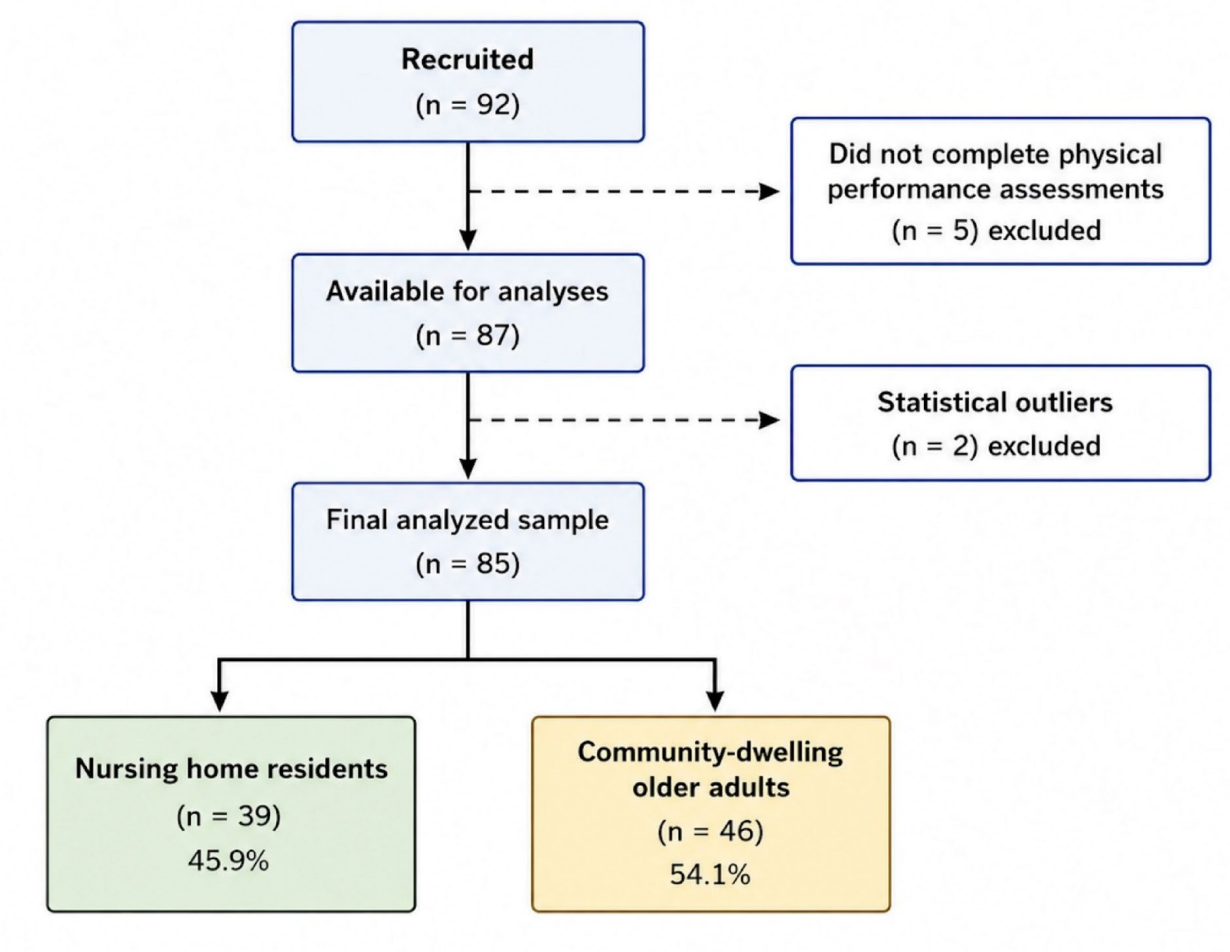
Flow diagram of participant selection and analysis.

The sample size of the study was calculated in the G*Power 3.1 analysis system. Since no similar study has been reported in the literature, a pilot study was planned. After 10 participants were included in each group, the effect size was calculated via the Purdue Pegboard total score. With an effect size of 0.637, 42 participants were included in each group to conduct the study with 80% power. Considering the possible loss of data, 46 participants were included in the groups. A systematic approach was followed for data cleaning and the management of missing values. Of the initial 92 participants recruited, 5 (5.4%) were unable to complete the physical tests. Little’s MCAR test indicated that the missing data were Missing Completely at Random, χ²(33) = 25.79, *p* = 0.810; therefore, listwise deletion was employed. Additionally, data were screened for univariate outliers. Two (2.2%) observations were identified as extreme values and excluded from the final analysis. These were defined a priori as values exceeding ± 3 standard deviations from the mean. To ensure methodological transparency and prevent bias, the identification and exclusion of outliers were conducted while blinded to group information. The final analysis was performed on 85 participants (n_1_ = 39 vs. n_2_ = 46) (Fig. 1). A post hoc power analysis was conducted based on the observed group means (Mean₁ = 31.41, SD₁ = 6.44; Mean₂ = 37.43, SD₂ = 8.34) and sample sizes (n₁ = 39, n₂ = 46) for the PPT total score. This analysis yielded an effect size (Cohen’s d) of 0.80. Using a two-tailed alpha of 0.05, the achieved power was approximately 98%, indicating sufficient power to detect the observed effect. Furthermore, all other parameters also achieved power above the expected 80%.

### Procedures

Within the scope of the study, after the demographic information of the older adults was recorded, hand grip strength was assessed with the Jamar hand dynamometer, and pinch strength was assessed with the Jamar digital pinch gauge. Manual dexterity was assessed with the Purdue Pegboard Test, and cognitive functions were assessed with the MoCA and Stroop T-Bag Form.

Socioeconomic status was determined based on participants’ self-reported monthly household income in Turkey. Income levels were categorized relative to the national minimum wage at the time of data collection^25^. Participants were classified as having low socioeconomic status if their monthly household income was below the national minimum wage, medium socioeconomic status if their income was between the minimum wage and twice the minimum wage, and high socioeconomic status if their income exceeded twice the national minimum wage. Regular exercise habits were classified based on participants’ self-reported weekly physical activity. Individuals were categorized as “exercising” if they reported engaging in structured physical activity (e.g., walking, group exercises, or strength training) for at least 30 min per day, 5 days per week, in accordance with international guidelines^26^.

Hand grip strength test: The hand grip strength assessment was performed via the Jamar hand dynamometer. The patient was seated upright, with the back supported and the hips leaning against the back of the chair, with the knee and elbow joints bent at a 90-degree angle. In addition, the arm should be free and parallel to the body, and the wrist should be kept in a neutral position without any deviation. The device’s grip was adjusted to two positions, which were the standard for all participants. Measurements were taken only with the participants’ dominant hand. The patient is asked to squeeze the dynamometer with maximal force. The measurement is repeated three times, each 10 s apart, and the final grip strength value is determined by averaging these three measurements^27^. The measured force is usually recorded in kilograms (kg)^9^.

Pinch meter: Pinch strength assessment was performed via the Jamar Digital Pinch Gauge. Before starting the study, the device was calibrated according to manufacturer standards by the Authorized Service, and the validity of the calibration was confirmed throughout the study. The position was the same as for the HGS. Three different grip types were evaluated in accordance with standard terminology: Lateral (Key) Pinch, Tip (Two-point) Pinch, and Palmar (Three-jaw Chuck) Pinch. The tip pinch was placed between the tips of the thumb and index finger; the lateral pinch was placed between the lateral surfaces of the thumb and index finger; and the palmar pinch was placed between the tips of the thumb, index finger, and middle finger. The patient was asked to squeeze the pinch meter with the maximum possible force. For each grip type, measurements were repeated three times at 10-second intervals, and the final force value was recorded in kilograms (kg) by averaging the three measurements^28,29^.

Purdue Pegboard Test (PPT): This test is used to objectively assess upper extremity functions, manual dexterity, and hand‒eye coordination. The PPT consists of a standard board with two rows of 25 holes and a box containing four holes. These containers contain metal pins, washers, and nuts. Participants were first instructed to place as many pins as possible into the holes in the board with their right hand within 30 s. After repeating the same test with their left hand, participants were asked to place the pins symmetrically using both hands simultaneously. Finally, they were instructed to make connections using pins, nuts, washers, and nuts for 60 s. Participants were asked to perform trials before the test period began in each condition. The test period began when they were ready. The timer continued without stopping in case of errors, such as dropping a piece. The number of pins placed in the first three tests and the number of all pins, washers, and nuts installed on the board in the last test were recorded^29,30^. These four separate scores were added to form a total test score for analysis.

Stroop T-Bag Form: This form assesses cognitive functions such as perceptual configuration and the ability to modulate responses under disruptive influences, information processing speed, and selective attention. In our study, we used the Stroop-TBAG version, standardized for the Turkish-speaking population by the Turkish Cognitive Research Group (TBAG) and whose validity/reliability has been established. The test was administered in a consistent and standardized environment for all participants. Standard room lighting was provided for environmental conditions, and the reading distance to the material was fixed at 40–50 cm. The test material was presented on cards measuring 14.0 × 21.5 cm per card and printed in 40-point Times New Roman font. In this five-part test, participants were asked to name colors written in black on a white background on the first card; colors written in a different color than their original color on the second card; colors of circles in different colors on the third card; colors of neutral words written in different colors on the fourth card; and colors of words written using the second card on the fifth card. Individuals’ completion times for the relevant task were recorded with a stopwatch for each of the five sections of the test. For analysis, a composite score was obtained by adding the five card times and dividing by five^31^.

**Fig. 2:**
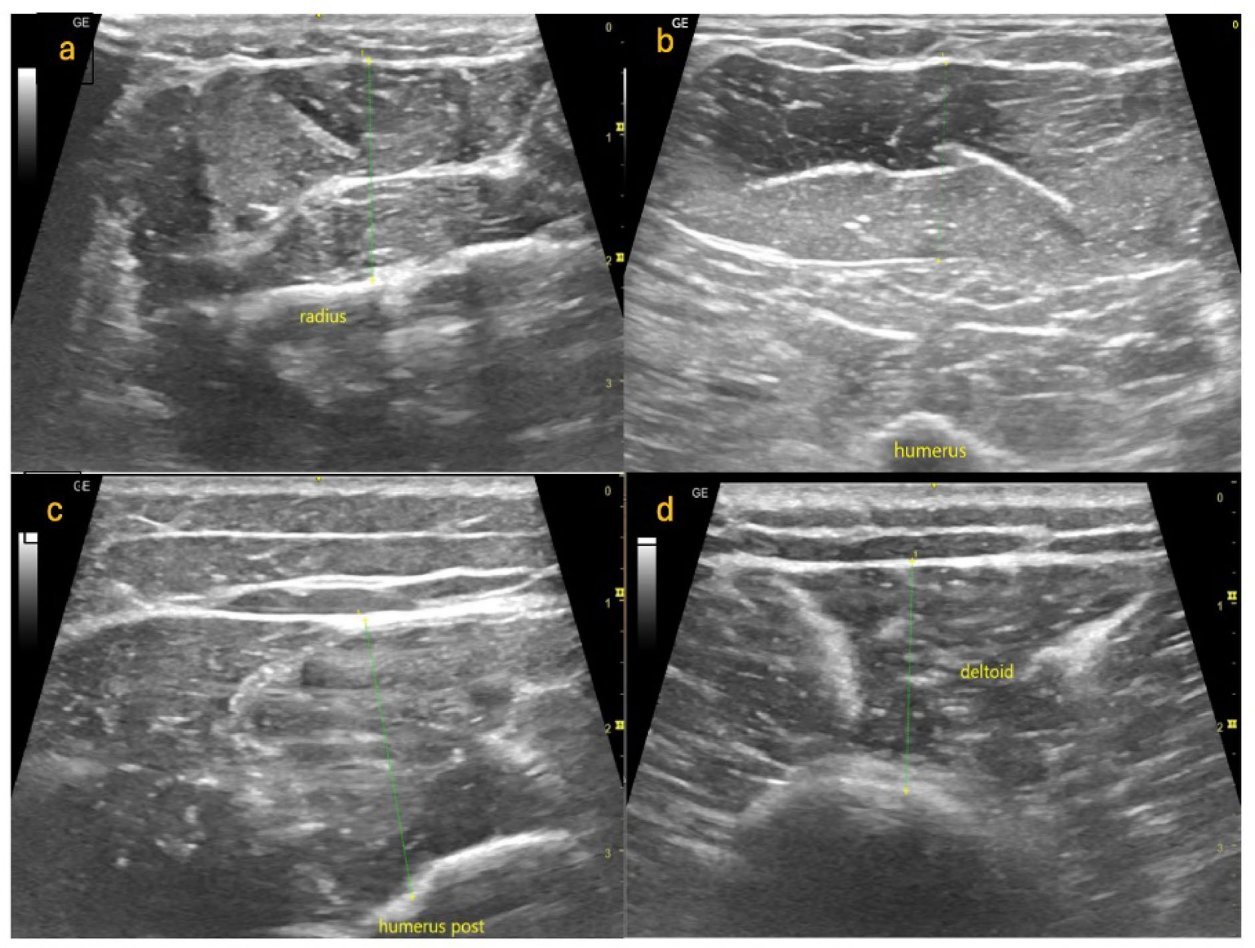
(**a**) Forearm, (**b**) Biceps Brachii, (**c**) Triceps Brachii, (**d**) Deltoid muscle thickness measurements on ultrasound image.

Ultrasonographic measurement: In our study, we used ultrasonography (US), a portable, cost-effective, and radiation-free method, to measure muscle thickness in the upper extremities (forearm, biceps brachii, triceps brachii, and deltoid). The literature reports that ultrasound is a valid and reliable measurement tool with high sensitivity and specificity for determining both regional and total muscle mass^32,33^. To obtain reliable and consistent measurements in our study, all ultrasonographic measurements were performed by the same experienced physiotherapist trained in musculoskeletal ultrasonography. Standardization procedures included the use of predefined anatomical landmarks, relaxed muscle positioning, minimal probe pressure using sufficient gel, and averaging of three repeated measurements for each muscle to reduce measurement variability. Measurements were made using linear probes (7 to 12 MHz) on a GE Versana^®^ active™ device. Gain was set to 60 and Depth was set to 4 cm for image optimization. All US images were taken with muscle tone relaxed, using ample gel to minimize probe pressure, and measurements were taken during the end-expiratory phase of respiration. The thickness was defined as fascia-to-fascia, with three repetitions performed for each muscle, and the arithmetic mean was used as the final value. Measurements were taken by placing the probe at the relevant sites on the dominant side with the participants comfortably seated on the examination table. Anatomical landmarks and measurement locations are as follows: Biceps brachii muscle^34^, two-thirds of the distance between the elbow fold and the tip of the acromion; Triceps brachii muscle^10^, at the posterior distal 60% of the distance between the acromion and the antecubital fossa; Deltoideus muscle^35^, at the midpoint of the joint between the lateral acromion edge and the deltoid tuberosity; and Forearm muscle^10^, at the proximal anterolateral 30% of the distance between the radial head and the styloid process. Muscles were measured in the axial view at the same point (Fig. 2).

### Statistical analysis

In this study, older adults were grouped according to their living environment (in the community and in a nursing home), and their demographic and clinical characteristics were analyzed in IBM SPSS Statistics 26. The assumption of normality of the data was comprehensively assessed using descriptive and inferential statistics, as well as visual methods (histograms and Q-Q plots), and homogeneity of variance was assessed using the Levene test. Age and BMI variables of the groups were compared using the Independent Sample T test, and categorical variables were compared using Fisher’s Exact Test.

To examine differences in cognitive function, handgrip strength, dexterity, and upper extremity muscle thickness between individuals living in the community and nursing homes, Analysis of Covariance (ANCOVA) was conducted to control for potential confounding factors known to influence cognitive and physical performance in older adults. A separate model was constructed for each dependent variable. The independent group variable was residence (living in the community vs. nursing home).

Age, education level, and socioeconomic status were used as confounding factors in the ANCOVA models to examine differences in Stroop and MoCA scores, which assess cognitive function, as these variables are widely recognized determinants of cognitive performance in older adults^36,37^. Age, BMI, and gender were used as confounding factors in the models for manual dexterity, grip strength, and upper extremity muscle thickness because these factors are known to influence muscle strength and physical performance^38–40^.

Prior to the analyses, assumptions of ANCOVA, including normality, homogeneity of variances, and homogeneity of regression slopes, were assessed. The homogeneity of regression slopes assumption was tested by examining the interaction terms between the group variable and each covariate, and all interaction terms were non-significant (*p* > 0.05), indicating that this assumption was met. A *p* < 0.05 level of statistical significance was used in all analyses. The adjusted means reported in the results correspond to the estimated marginal means derived from the ANCOVA models.

## Results

A total of 85 older adults, 39 living in nursing homes and 46 community-dwelling adults, participated in our study. As indicated in Table 1, there were no statistically significant differences between the participant groups in terms of age, body mass index, sex, educational level, socio-economic status or regular exercise habits.

**Table 1:** Demographic and clinical characteristics of the older adults.

		**Nursing home resident** **(n=39)**		**Community-Dwelling** **(n=46)**		**p** ^a^
		**Mean (SD)**	** (IQR)**	Mean (SD)	~** (IQR)**	
Age (year)		72.87 (4.11)	74 (8)	71.52 (3.79)	72 (7.25)	0.122
BMI (kg/m^2^)		25.44 (1.22)	25.64 (1.77)	25.88 (1.02)	26.1 (1.44)	0.080
		**n**	**%**	**n**	**%**	**p** ^b^
Gender	Female	24	61.5	22	47.8	0.275
	Male	15	38.5	24	52.2	
Regular exercise habits	Exercising	28	71.8	26	56.5	0.178
	None	11	28.2	20	43.5	
Educational level	Primary school	4	10.3	3	6.5	0.910
	Middle school	7	17.9	8	17.4	
	High school	10	25.6	11	23.9	
	Higher education	18	46.2	24	52.2	
Socio-economic status	Low	2	5.1	6	13.0	0.459
	Medium	28	71.8	30	65.2	
	High	9	23.1	10	21.7	

a: Independent Samples Test, b: Fisher’s Exact Test, SD: Standard Deviation, ~(IQR): Median (Inter Quantile Range), kg: kilogram, m: meter, n: Number of patients, BMI: Body Mass Index.

After controlling for confounding factors (age, BMI, and gender) (Table 2), statistically significant differences were observed between nursing home residents and community residents in terms of upper extremity muscle thickness, grip strength, and manual dexterity.

**Table 2:** Results of ANCOVA comparing upper extremity muscle thickness, grip strength, and manual dexterity between groups, adjusting for age, body mass index, and gender.

	Group	Unadjusted mean (SD)	Unadjusted difference mean [95% CI]	Adjusted mean (SE)	95% CI for the Adjusted Mean	Adjusted difference mean [95% CI]	F	p-value	η²ₚ
US – Forearm (mm)	Nursing Home Resident	13.39 (3.29)	−1.24 [−2.62, 0.14]	13.20 (0.39)	12.42–13.98	−1.36 [−2.42, −0.30]	6.55	0.012*	0.077
	Community-Dwelling	14.63 (3.11)		14.56 (0.35)	13.86–15.26				
US - Biceps Brachii (mm)	Nursing Home Resident	14.33 (3.84)	−1.90 [−3.46, −0.34]	14.34 (0.59)	13.16–15.52	−1.72 [−3.33, −0.11]	4.57	0.036*	0.055
	Community-Dwelling	16.23 (3.36)		16.06 (0.53)	15.01–17.12				
US – Triceps Brachii (mm)	Nursing Home Resident	20.29 (4.72)	−3.01 [−5.01, −1.02]	20.09 (0.73)	18.63–21.55	−3.06 [−5.05, −1.07]	9.41	0.003*	0.106
	Community-Dwelling	23.31 (4.48)		23.25 (0.65)	21.84–24.46				
US – Deltoid (mm)	Nursing Home Resident	12.86 (5.12)	−2.26 [−4.00, −0.51]	13.11 (0.66)	11.78–14.43	−1.90 [−3.71, −0.10]	4.44	0.038*	0.053
	Community-Dwelling	15.12 (2.78)		15.02 (0.59)	13.83–16.20				
HGS (kg)	Nursing Home Resident	18.64 (6.42)	−3.90 [−6.52, −1.29]	17.82 (0.93)	15.95–19.68	−4.96 [−7.50, −2.42]	15.09	< 0.001*	0.160
	Community-Dwelling	22.54 (5.69)		22.78 (0.84)	21.10–24.45				
PS - Lateral (kg)	Nursing Home Resident	11.03 (2.41)	−2.05 [−3.26, −0.84]	10.88 (0.46)	9.95–11.81	−2.22 [−3.49, −0.95]	12.21	0.001*	0.134
	Community-Dwelling	13.08 (3.07)		13.10 (0.42)	12.27–13.94				
PS - Tip (kg)	Nursing Home Resident	8.81 (3.29)	−2.01 [−3.52, −0.51]	8.88 (0.54)	7.79–9.97	−1.90 [−3.39, −0.42]	6.52	0.013*	0.076
	Community-Dwelling	10.83 (3.61)		10.78 (0.49)	9.80–11.76				
PS - Palmar (kg)	Nursing Home Resident	9.83 (2.86)	−2.06 [−3.49, −0.62]	9.74 (0.53)	8.67–10.81	−2.21 [−3.66, −0.75]	9.17	0.003*	0.104
	Community-Dwelling	11.89 (3.64)		11.95 (0.48)	10.99–12.91				
PPT - Total	Nursing Home Resident	31.41 (6.44)	−6.02 [−9.21, −2.82]	30.98 (1.13)	28.73–33.22	−6.39 [−9.46, −3.33]	17.25	< 0.001*	0.179
	Community-Dwelling	37.43 (8.34)		37.37 (1.01)	35.35–39.40				

*: *p* < 0.05, SD: Standard Deviation, SE: Standard Error, CI: Confidence Interval, mm: millimeter, US: Ultrasonography HGS: Hand Grip Strength, PS: Pinch Strength, PPT: Purdue Pegboard Test.

Upper extremity muscle thickness measurements showed significant differences in favor of community residents. Triceps brachii muscle thickness was 15.2% lower in nursing home residents compared to the community group (F = 9.410, *p* = 0.003, η²*p* = 0.106). Similarly, muscle thicknesses of the biceps brachii (F = 4.570, *p* = 0.036, η²*p* = 0.055), deltoid (F = 4.440, *p* = 0.038, η²*p* = 0.053), and forearm (F = 6.55, *p* = 0.012, η²*p* = 0.077) were 12%, 14.5%, and 10.3% greater, respectively, in community residents compared to nursing home residents (Table 2).

Similar strong results were obtained in grip and pinch strength measurements. HGS was one of the parameters showing the strongest difference between the groups (F = 15.09, *p* < 0.001, η²*p* = 0.160), with community residents having 27.8% greater grip strength than nursing home residents. Significant differences were also observed in pinch strength measurements. Lateral grip strength (F = 12.210, *p* = 0.001, η²*p* = 0.134), tip grip strength (F = 6.52, *p* = 0.013, η²*p* = 0.076), and palmar grip strength (F = 9.17, *p* = 0.003, η²*p* = 0.104) were 20.4%, 21.4%, and 22.7% higher in community-dwelling individuals, respectively (Table 2).

Finally, the Purdue Pegboard Test (PPT) total score, a measure of manual dexterity, showed a strong difference based on living environment (F = 17.25, *p* < 0.001, η²*p* = 0.179). The adjusted PPT total score for individuals living in nursing homes was 20.6% lower than for those living in the community, indicating significantly poorer manual dexterity in the nursing home setting. Adjusted mean differences and 95% Confidence Intervals (CI) are presented in detail in Table 2.

In comparing the cognitive functions of individuals living in nursing homes with those living in the community, age, education level, and socioeconomic status were controlled for as confounding factors (Table 3). The mean adjusted Stroop test completion time for individuals living in the nursing home was significantly higher than for those living in the community (F = 8.96, *p* = 0.004, η²*p* = 0.121). This finding indicates that the nursing home group completed the Stroop test 23.6% slower (lower performance) than the community group (Table 3).

**Table 3:** Results of ANCOVA comparing cognitive functions between groups, adjusting for age, educational level, and socio-economic status.

	Group	Unadjusted mean (SD)	Unadjusted difference mean [95% CI]	Adjusted mean (SE)	95% CI for the Adjusted mean	Adjusted difference mean [95% CI]	F	p-value	η²ₚ
MoCA	Nursing Home Resident	24.30 (1.34)	−0.58 [−1.21, 0.04]	23.98 (0.31)	23.35–24.61	−1.27 [−2.10, −0.43]	4.47	0.038*	0.064
	Community-Dwelling	24.89 (1.53)		25.25 (0.27)	24.70–25.79				
Stroop composite score (sec)	Nursing Home Resident	22.40 (5.29)	2.69 [0.84, 4.55]	23.77 (0.92)	21.92–25.62	4.53 [2.07, 6.99]	8.96	0.004*	0.121
	Community-Dwelling	19.70 (3.20)		19.23 (0.80)	17.62–20.84				

*: *p* < 0.05, SD: Standard Deviation, SE: Standard Error, CI: Confidence Interval, sec: second, MoCA: Montreal Cognitive Assessment Scale.

A significant effect of living environment was also found on MoCA scores (F = 4.47, *p* = 0.038, η²*p* = 0.064). The mean adjusted MoCA for individuals living in the community was statistically significantly higher than for those living in the nursing home. This suggests that the cognitive performance of nursing home residents was approximately 5.3% lower than that of those living in the community. These results suggest that, even when controlling for confounding factors, elderly individuals living in nursing homes exhibit lower cognitive function than their community-dwelling peers. Adjusted mean differences and 95% Confidence Intervals (CI) are presented in detail in Table 3.

## Discussion

To better understand the effects of environmental factors on functionality, our study aimed to compare the effects of different living environments on upper extremity muscle thickness, grip strength, manual dexterity, and cognitive functions. Consistent with our expectations, our findings revealed that nursing home residents performed significantly lower on all these parameters than community-dwelling individuals. These findings suggest that living environment may play an important role in functional capacity during aging. While general exercise habits appear similar between the two groups, the significant differences observed in outcomes may be related to the decline in functional capacity not only with exercise frequency but also with the more complex and indirect interaction of living conditions and social resources. Community-dwelling individuals benefit from the high levels of security, belonging, and psychosocial comfort provided by established and familiar living environments^7^. More importantly, the mental and physical demands of daily responsibilities such as housework, shopping, and gardening keep individuals engaged in a continuous cycle of physical and cognitive activity. This constant, natural physical engagement (physical activity, distinct from formal exercise) is critical for maintaining muscle mass, dexterity, and daily cognitive function. On the other hand, while institutional health management for individuals living in nursing homes is optimized, their responsibilities for daily household chores are minimized. However, the removal of household responsibilities and daily workload may lead to a decrease in the individual’s natural physical capacity used during activities of daily living (ADL). Despite the ease of health management and social opportunities offered in institutional care settings such as nursing homes, the reduction in complex daily tasks may be associated with lower total daily energy expenditure and routine cognitive load^6,7^ In this study, the lower physical and cognitive performance observed among nursing home residents may be associated with reduced exposure to the natural daily functional demands required for independent living.

In our study, we used ultrasonography (US), which has been reported to have good intrarater and interrater reliability and test-retest reliability in the evaluation of upper extremity muscles in older adults^32,41^. US also offers significant advantages in field studies and clinical settings because it is a portable, cost-effective, and radiation-free imaging tool. Beyond these practical benefits, US-measured muscle thickness has been demonstrated in the literature to have high sensitivity and specificity in assessing both regional and total muscle mass^32,33^. There are no studies in the literature comparing upper extremity muscle thickness with US in elderly adults living in nursing homes or in the community. In studies examining upper extremity muscle thickness with US, groups were generally made according to personal variables such as sex, age, presence of sarcopenia, or the relationship between muscle thickness and demographic and clinical data. It has been reported that muscle thickness decreases with age, varies according to sex, is lower in sarcopenic individuals, and is related to age and body mass index^10,42–44^. The mean biceps brachii muscle thickness reported in these studies was consistent with the mean age of community-dwelling individuals in our study, while the mean for male participants was higher. Similarly, the mean for community-dwelling individuals was similar to the mean for sarcopenic and nonsarcopenic individuals. However, the mean for individuals living in nursing homes was lower than the mean reported in the literature. Consistent with our expectations, the results of our study revealed that the average muscle thickness of all upper extremities in elderly individuals residing in nursing homes was significantly lower than in individuals living in the community. This difference may be related to lifestyle changes associated with institutionalization. Unlike individuals living in the community, individuals living in nursing homes, as a result of institutionalization, abandon daily household responsibilities such as cooking, cleaning, and shopping, which require active use of the upper extremities. The removal of these daily responsibilities may reduce the frequency and intensity of upper extremity muscle use. This reduced upper extremity use during daily activities may partially explain the differences observed between the groups in this study.

Although a higher proportion of nursing home residents reported participation in regular exercise compared to community-dwelling older adults, this was not accompanied by higher muscle mass. This finding may be related to differences in physical activity between the two settings. While exercise participation in nursing homes may be more structured, community-dwelling older adults may engage in a broader range of daily life activities (ADLs), such as walking and household tasks, which could contribute to overall muscle preservation^45^. Therefore, these results should be interpreted by considering not only reported exercise participation but also habitual daily physical activity.

In addition to reduced mechanical loading related to daily activity patterns, nutritional status may also be associated with the observed differences in muscle thickness, as protein and energy intake are well-established determinants of muscle mass and sarcopenia in older adults^46^. Although nursing home residents generally receive standardized meals, individual nutritional intake may be influenced by factors such as appetite loss, comorbidities, or feeding assistance needs. Conversely, community-dwelling older adults may have greater autonomy over dietary choices but can also be exposed to nutritional inadequacy or imbalance. Therefore, nutritional factors should be considered when interpreting the present findings, particularly in institutionalized versus community-living older populations. Future studies should include standardized nutritional assessment tools, such as the Mini Nutritional Assessment (MNA), to better clarify the potential role of nutritional status in these relationships.

The prevalence of sarcopenia is reported to be greater in older adults, especially those living in nursing homes or hospitals, than in adults living in the community^17–19^. These studies reported that differences in muscle mass and physical performance of elderly individuals in nursing homes and at home may be related to low physical activity or prolonged bed rest, depressive mood and social isolation. Hand grip strength is frequently used as an indicator of general muscle strength in older adults, especially in the diagnosis of sarcopenia^9,47^. It is known that hand grip strength decreases in older adults. It has been reported in the literature that hand grip strength is related to physical and cognitive status as well as daily living activities^9,48,49^. However, few studies have compared the hand grip strength of older adults living in nursing homes and in the community. A study conducted by Roberts et al^20^. reported that the grip strength of elderly individuals in rehabilitation and nursing home environments was lower than that of those living at home. The study conducted by Taketomi et al^21^. aimed to compare the strength of the right dominant hand between community residents and institutional residents via a hand dynamometer. The results of this study revealed that the grip strength and lateral pinch strength of elderly individuals were affected by environmental conditions and were lower in nursing home residents than in community residents. Our study results support the literature, and it was observed that older adults living in nursing homes had lower values for hand grip strength, which is an indicator of general muscle strength and sarcopenia.

Pinch grip strength reportedly decreases with age^28,50^, and community dwellers have better lateral pinch strength than institutional dwellers do^21^. In our study, lateral, palmar and tip compression strengths were evaluated. All three compression strengths were consistent with normative values in older adults living in the community but lower than normative values in older adults living in nursing homes.

The literature indicates that manual dexterity may decrease with age. The Purdue Pegboard Test is commonly used by health professionals to measure decreases in manual dexterity^30^. In our study, it was observed that elderly adults living in the community had values similar to the normative values of the Purdue Pegboard Test presented in the literature^30,51,52^, whereas those of adults living in nursing homes were below the normative values. In addition, the literature suggests that manual asymmetry decreases with age due to a decrease in the performance of the dominant hand^53^. In our study, the dominant and nondominant hand performances were symmetrical in both groups, similar to the literature.

The literature demonstrates that the daily responsibilities assumed by older adults vary depending on the environment they live in. While community-dwelling individuals residing in their own homes continue to assume the various responsibilities required by the home environment, those living in nursing homes experience significantly reduced domestic responsibilities^6,7^. It is highly likely that individuals living in nursing homes become dependent on others for daily household tasks that require active use of the upper extremities, such as cooking or cleaning. This reduction in the natural, necessary physical demands and environmental complexity encountered by community-dwelling older adults may be associated with the significantly lower muscle thickness, grip and pinch strength, and manual dexterity observed in the nursing home group.

Cognitive impairment is a common disorder in older adults resulting from conditions associated with aging^54^. Although cognitive impairment can be affected by environmental factors^55–57^, only one study has compared the cognitive status of older adults living in nursing homes and in the community. In the study conducted by Setiyani et al.^23^, assessed cognitive status using the Mini Mental State Examination (MMSE) and found that Indonesian older adults in nursing homes had lower mean scores than those living in the community. In our study, MoCA scores and Stroop T-Bag card times were significantly lower in nursing home residents. The observed cognitive differences between the two groups may be attributable to several critical factors common in the nursing home environment. The lack of complex daily activities may play a significant role. In a community setting, older adults are often required to engage in a wide variety of cognitively stimulating tasks, such as meal planning, managing finances, and navigating public spaces for shopping or appointments. In contrast, the nursing home environment, while providing basic care, often reduces or eliminates these daily responsibilities. Depression is frequently observed in nursing home residents because of environmental changes and loneliness, and it has been reported that increased depressive symptoms may also cause cognitive impairment^55–57^. Although depression levels were not directly measured in our study, this cognitive difference in individuals living in nursing homes may be a reflection of the environmental factors to which individuals are exposed and the cognitive impairments associated with depression, which are frequently mentioned in the relevant literature. This interpretation is consistent with a 22-year follow-up study reporting greater cognitive decline among older adults entering institutional care compared with those living in the community^58^. However, these findings should be interpreted considering that the study sample consisted of relatively high-functioning older adults and may not fully represent more vulnerable nursing home residents with advanced cognitive or physical impairments.

Our study is the first to compare the upper extremity muscle thickness and manual dexterity of individuals living in a nursing home with those of individuals living in a community. In addition, the evaluation of all pinch strengths, the use of the MoCA, which is a more comprehensive test than the MMSE, and the Stroop T-Bag Form, which evaluates more specific functions, are the strengths of our study. The limitations of our study include the lack of long-term follow-up, the lack of use of functional independence measures, and the lack of a comprehensive evaluation of physical activity levels. In addition, several potentially important confounding factors were not directly assessed in this study, including nutritional status using standardized tools (e.g., Mini Nutritional Assessment, dietary assessment, or biochemical markers), depressive symptoms, activities of daily living (ADL), and objectively measured physical activity levels. These factors may have influenced muscle thickness, strength, manual dexterity, and cognitive performance outcomes. Additionally, intra-rater reliability was not formally assessed, and assessor blinding was not feasible due to the study setting, which may have introduced measurement bias. Another limitation is that multiple statistical comparisons were conducted across several outcome measures, which may increase the risk of Type I error. Although the observed effect sizes were generally moderate to large and consistent across related outcomes, the findings should nevertheless be interpreted with caution. In addition, the external validity of the findings may be limited because the study sample consisted of relatively high-functioning older adults who met specific inclusion criteria (e.g., MoCA ≥ 21, independent standing ability, absence of severe neurological or sensory impairments). Therefore, the results may not be generalizable to more vulnerable nursing home residents with advanced cognitive or physical impairments.

## Conclusion

In this study, older adults living in nursing homes were observed to have lower upper extremity muscle thickness, handgrip strength, pinch strength, manual dexterity, and cognitive performance compared with community-dwelling older adults. These findings, along with the reduced daily responsibilities such as cooking, housekeeping, and shopping in the nursing home environment, may reflect differences in general living conditions, social resources, and environmental factors between the groups. These findings suggest that maintaining upper extremity muscle thickness and strength, manual dexterity, and cognitive functions may be important for older adults living in nursing homes. Our study results shed light on further studies that will focus on cognitive functions and upper extremity functions and long-term follow-up. These cross-sectional findings may contribute to future studies investigating functional and cognitive differences in nursing home residents.

## Data Availability

The datasets generated and analyzed during the current study are not publicly available for ethical reasons but are available from the corresponding author upon reasonable request.
